# Alteration of mitochondrial protein succinylation against cellular oxidative stress in cancer

**DOI:** 10.1186/s40779-022-00367-2

**Published:** 2022-02-04

**Authors:** Jing Zhang, Zi-Qin Han, Yang Wang, Qing-Yu He

**Affiliations:** 1 Department of Radiology, The First Affiliated Hospital of Jinan University, Guangzhou, 510627 China; 2 MOE Key Laboratory of Tumor Molecular Biology and Key Laboratory of Functional Protein Research of Guangdong Higher Education Institutes, Institute of Life and Health Engineering, College of Life Science and Technology, Jinan University, Guangzhou, 510632 China; 3 MOE Key Laboratory of Tumor Molecular Biology, The First Affiliated Hospital of Jinan University, Guangzhou, 510627 China

**Keywords:** Mitochondria, Succinylation, Phosphorylation, Glutaminase

Dear Editor,

Lysine residue succinylation is a novel post-translational modification that recently attracted extensive attention. Succinylation is achieved by non-enzymatic processes or by a series of enzymes [like p300, lysine acetyltransferase 2A (KAT2A)] that transfer the succinyl groups from succinyl-coenzyme A (CoA) to the specific lysine, modulating protein function in various physiological processes [1]. As a high-energy metabolite, succinyl-CoA is mainly produced within the mitochondrial matrix and peroxisomes. Its high-rate generation in the tricarboxylic acid (TCA) cycle and its impermeability across the mitochondrial inner membrane (due to its negative charge property) enhance succinyl-CoA accumulation within mitochondria. It is therefore not surprising that mitochondrial proteins have a higher potential to be succinylated, although succinylation has been reported to be accumulated at transcriptional starting sites, modulating histones to regulate gene expression. Being a key component of the TCA cycle, the α-ketoglutarate dehydrogenase (α-KGDH) complex as the key source of succinyl-CoA has been found to interact with KAT2A in the nucleus for mediating histone succinylation on H3K79. Notably, only 1.0–1.6% of the α-KGDH complex is located in the nucleus, which consequently contributes to histone succinylation [2].

Succinyl-CoA ligase (SCL) located in the mitochondrial matrix, is an important player in succinylation. The ligase works as a heterodimer consisting of the succinate-CoA ligase GDP/ADP-forming subunit α/β (SUCLG1/2) and succinate-CoA ligase ADP-forming subunit β (SUCLA2), with its deficiency enhancing the accumulation of succinylated proteins. Mutations of SCL in patients, including Asp333Gly, Arg407Trp and 13q14 deletion, inhibit SCL activity, resulting in an increase of succinyl-CoA level [3]. Tong et al. [4] recently reported that the phosphorylation of SUCLA2, but not SUCLG2, detached its interaction from glutaminase (GLS), promoting its succinylation, and consequently enhancing the GLS activity in pancreatic ductal adenocarcinoma (PDAC) tissues. This study provides essential evidence for stress-induced succinylation in cancer to accommodate increased metabolic burden.

Similar to other tumors, PDAC cells prefer glutamine for anabolic processes to sustain proliferation. Being the first enzyme of glutamine catabolism, GLS mainly resides inside mitochondria to catalyze glutamine into glutamate and ammonia. It is upstream-regulated by c-Jun, which directly binds to the GLS promoter region, to promote the progression of breast cancer. In acute myeloid leukemia, GLS inhibition impairs cellular antioxidant glutathione (GSH) production and the mitochondrial redox state [5]. GLS inhibition is therefore considered as a potential strategy for cancer therapeutics. Tong et al. [4] first confirmed that GLS was upregulated in human PDAC tissues, with PDAC cells exhibiting highly elevated GLS activity, when compared with normal pancreatic duct epithelial cells. GLS was found to undergo succinylation at the evolutionarily conserved K311. A hydrogen bond is formed between succinylated K311 and H475 from adjacent monomers at the interface of GLS, resulting in the subsequent enhancement of GLS oligomerization and activity.

Succinyl-CoA synthetase/SUCLA2 was found to interact and co-localize with GLS, which acted as a critical succinylation regulator to remove the succinyl-group from GLS-K311. Upon H_2_O_2_ stimulation, p38 is triggered to phosphorylate SUCLA2 at S79, which promotes the dissociation of SUCLA2 from GLS, leading to an increase of local succinyl-CoA levels in close proximity to GLS and ultimately promoting GLS succinylation/polymerization (Additional file 1: Fig. S1). In the experiments, precise quantification of the succinyl-CoA level in mitochondria will better support this conclusion. In addition, whether p38 translocates into the mitochondria for SUCLA2 phosphorylation remains to be further elucidated. Moreover, they found that K311-succinylation of GLS promoted glutamine metabolism, leading to the production of nicotinamide adenine dinucleotide phosphate (NADPH) and GSH, which were essential to offset reactive oxygen species (ROS)-cytotoxicity (Additional file 1: Fig. S1). In addition, analyses of clinical samples revealed that SUCLA2-S79 phosphorylation and GLS-K311 succinylation levels were positively correlated with each other and were also positively correlated with the clinical stage and poor prognosis of patients with PDAC.

Increased TCA metabolites in mitochondria, including α-ketoglutarate and succinic acid, have been shown to proceed with novel post-translational modifications on lysine (Additional file 1: Fig. S1). As observed in PDAC, the interplay between post-translational modifications results in the activation of p38-SUCLA2-GLS-NADPH-GSH signaling, which contributes to cancer survival and drug-resistance. More efforts are needed to develop dual-target inhibitors of SUCLA2-S79 and GLS-K311 for clinical cancer therapy, which has the potential to lower therapeutic dosages and avoid drug resistance.

## Supplementary Information

**Additional file 1. Fig. S1:** Mechanism of GLS succinylation against cellular oxidative stress in cancer. GLS is highly expressed in cancer, which is regulated upstream by c-Jun, that directly binds to the GLS promoter region. GLS can interact and colocalize with SUCLA2 in mitochondria. Upon oxidative stress, SUCLA2 phosphorylated by p38 dissociates from GLS, resulting in enhanced GLS succinylation and activity. Activated GLS increases glutaminolysis and then supplies α-ketoglutarate to TCA cycle, which is subsequently metabolized to succinyl-CoA. Succinyl-CoA-dependent GLS succinylation leads to increased production of NADPH and GSH, which neutralize ROS induced by oxidative stress and promotes tumor growth. GLS glutaminase, SUCLA2 succinate-CoA ligase ADP-forming subunit β, CoA coenzyme, TCA tricarboxylic acid, NADPH nicotinamide adenine dinucleotide phosphate, NADP^+^ oxidized form of NADPH, GSH glutathione, GSSG glutathione disulfide, ROS reactive oxygen species, P phosphate group, Succ succinylate group.

## Data Availability

Not applicable.

## Abbreviations

α-KGDH: α-Ketoglutarate dehydrogenase
CoA: Coenzyme
GLS: Glutaminase
GSH: Glutathione
KAT2A: Lysine acetyltransferase 2A
NADPH: Nicotinamide adenine dinucleotide phosphate
PDAC: Pancreatic ductal adenocarcinoma
ROS: Reactive oxygen species
SCL: Succinyl-CoA ligase
SUCLA2: Succinate-CoA ligase ADP-forming subunit β
SUCLG1/2: Succinate-CoA ligase GDP/ADP-forming subunit α/β
TCA: Tricarboxylic acid
