# Supplementary figures and images for: Alteration of mitochondrial protein succinylation against cellular oxidative stress in cancer
Source: Mil Med Res. 2022 Feb 4;9:6. doi: 10.1186/s40779-022-00367-2 (PMC8815146; doi:10.1186/s40779-022-00367-2)

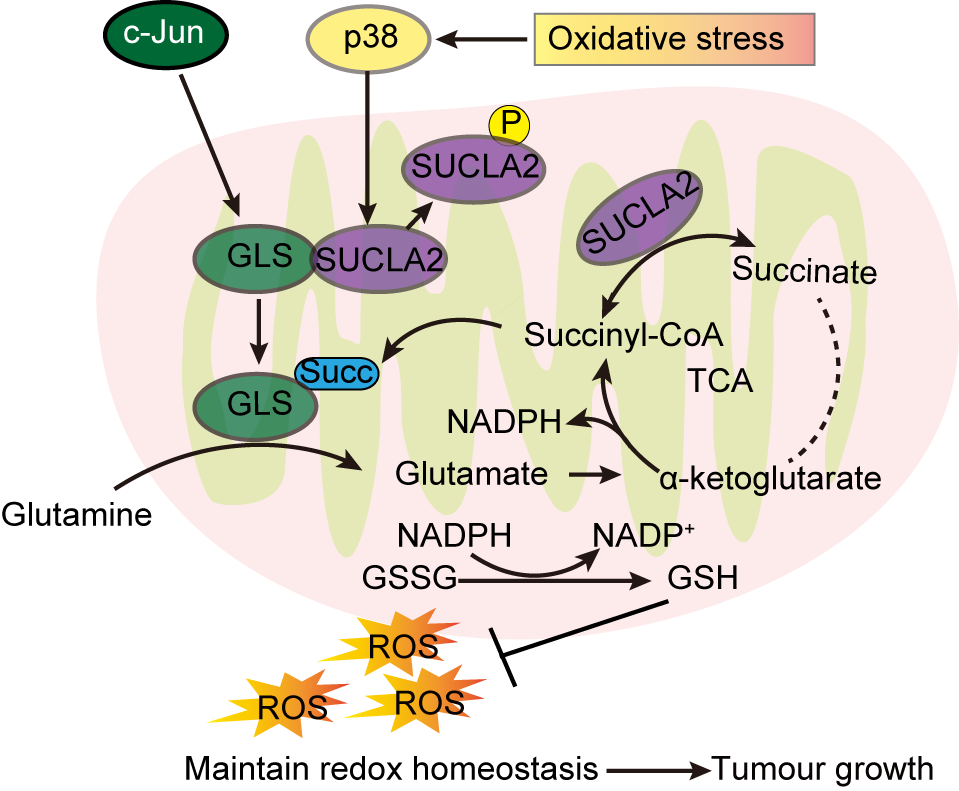

Supplement: Supplementary file 1 — Additional file 1. Fig. S1: Mechanism of GLS succinylation against cellular oxidative stress in cancer. GLS is highly expressed in cancer, which is regulated upstream by c-Jun, that directly binds to the GLS promoter region. GLS can interact and colocalize with SUCLA2 in mitochondria. Upon oxidative stress, SUCLA2 phosphorylated by p38 dissociates from GLS, resulting in enhanced GLS succinylation and activity. Activated GLS increases glutaminolysis and then supplies α-ketoglutarate to TCA cycle, which is subsequently metabolized to succinyl-CoA. Succinyl-CoA-dependent GLS succinylation leads to increased production of NADPH and GSH, which neutralize ROS induced by oxidative stress and promotes tumor growth. GLS glutaminase, SUCLA2 succinate-CoA ligase ADP-forming subunit β, CoA coenzyme, TCA tricarboxylic acid, NADPH nicotinamide adenine dinucleotide phosphate, NADP+ oxidized form of NADPH, GSH glutathione, GSSG glutathione disulfide, ROS reactive oxygen species, P phosphate group, Succ succinylate group. [file 40779_2022_367_MOESM1_ESM.tif]
